# Current Understanding of the Role of Cytoskeletal Cross-Linkers in the Onset and Development of Cardiomyopathies

**DOI:** 10.3390/ijms21165865

**Published:** 2020-08-15

**Authors:** Ilaria Pecorari, Luisa Mestroni, Orfeo Sbaizero

**Affiliations:** 1 Department of Engineering and Architecture, University of Trieste, 34127 Trieste, Italy; ipecorari@units.it; 2 University of Colorado Cardiovascular Institute, University of Colorado Anschutz Medical Campus, Aurora, CO 80045, USA; LUISA.MESTRONI@cuanschutz.edu

**Keywords:** cytoskeleton, actin-binding proteins, cross-linkers, alpha-actinin 2, filamin C, dystrophin, cardiomyopathies

## Abstract

Cardiomyopathies affect individuals worldwide, without regard to age, sex and ethnicity and are associated with significant morbidity and mortality. Inherited cardiomyopathies account for a relevant part of these conditions. Although progresses have been made over the years, early diagnosis and curative therapies are still challenging. Understanding the events occurring in normal and diseased cardiac cells is crucial, as they are important determinants of overall heart function. Besides chemical and molecular events, there are also structural and mechanical phenomena that require to be investigated. Cell structure and mechanics largely depend from the cytoskeleton, which is composed by filamentous proteins that can be cross-linked via accessory proteins. Alpha-actinin 2 (ACTN2), filamin C (FLNC) and dystrophin are three major actin cross-linkers that extensively contribute to the regulation of cell structure and mechanics. Hereby, we review the current understanding of the roles played by ACTN2, FLNC and dystrophin in the onset and progress of inherited cardiomyopathies. With our work, we aim to set the stage for new approaches to study the cardiomyopathies, which might reveal new therapeutic targets and broaden the panel of genes to be screened.

## 1. Introduction

Cardiovascular disorders (CVDs) are a leading cause of death globally and inherited cardiac diseases represent an important epidemiological entity, with combined estimated prevalence of 3% in the general population [1]. Despite tremendous advancements in cardiomyopathy research, diagnostic and therapeutic challenges are not uncommon. By way of example, life-threatening arrhythmias due to arrhythmogenic cardiomyopathy can manifest earlier than the development of clinical structural heart disease, resulting in a late—or even post-mortem—diagnosis [2]. On the other hand, available treatments consist only of palliative pharmacological care or invasive therapy [3]. Over the years, it has become increasingly clear that knowledge of the structural and mechanical properties of the heart are requisite to understand the causes and the consequences of CVDs, since these properties are pivotal determinants of the overall heart function. Biological systems, like the heart, are constructed via bottom-up processes and the organization of elements across length scales determines the properties of the whole system [4]. Therefore, understanding events occurring at molecular and cellular scale can improve our knowledge of the whole heart, in health and disease. When focusing on the cell, the cytoskeleton is recognized as a major player in regulating structural and mechanical properties. First, it provides the cell with both scaffolding and signaling functions. Like the steel framework of a high-rise building, it withstands the range of external and internal forces; simultaneously, it behaves like a highway, by facilitating the delivery of cargos from one point to another. Second, cytoskeleton generates, transmits and responds to forces and it controls the physical properties of the cell [5]. The cytoskeleton is a dynamic network of filamentous proteins, mostly microfilaments (also known as actin filaments), microtubules and intermediate filaments. Cytoskeletal filaments do not act alone but synergistically with many other accessory (or ancillary) proteins (APs). In particular, cytoskeletal filaments can be cross-linked to similar filaments and to membranes through these APs. Among cross-linking APs are actin-binding and crosslinking proteins, which are required for the formation, organization and customized functioning of the actin cytoskeleton. Whilst actin filaments alone are less rigid than microtubules, the presence of actin cross-linkers supports the assembly of highly organized and stiff structures, like isotropic networks, bundled networks and branched networks [5]. Besides morphological features (filament length, bundle thickness, mesh size), the mechanical properties of actin networks are therefore tuned by cross-linkers and their binding strength.

Given the relevance of cross-linkers in determining the physical properties of a cell, we hereby review the cases of inherited cardiomyopathies caused—or likely caused—by aberrant actin-binding and crosslinking proteins, namely alpha-actinin 2 (ACTN2), filamin C (FLNC) and dystrophin. A schematic overview of the main cardiomyopathies considered in this review is provided in Table 1; further details on prevalence, typical clinical presentation and so forth, are available in Reference [2]. 

We aim to collect the current understanding on the onset and progress of cardiomyopathies promoted by abnormal actin cross-linkers. We will focus on the structure of these cross-linkers, highlighting differences and similarities. To the best of our knowledge, we will describe the pathologic cardiac phenotypes associated so far with mutations in these cross-linkers, underlying possible overlaps or predominant characteristics. Our work could hopefully represent the impetus for studying novel diagnostic approaches or targeted therapies based on cross-linkers. 

## 2. Alpha-Actinin 2

Alpha-actinins are a family of actin-binding proteins (ABPs) and actin crosslinking proteins, belonging to the spectrin superfamily [13]. In particular, alpha-actinins are crucial to form actin bundles. In mammals, there are 4 alpha-actinin genes, coding for as many proteins. There are 2 muscle (ACTN2 and ACTN3) and 2 non-muscle isoforms (ACTN1 and ACTN4) [14]. Among the muscle isoforms, ACTN3 is expressed solely in skeletal muscle, whereas ACTN2 is expressed in both skeletal and cardiac muscle [15]. Among skeletal muscle isoforms, ACTN2 is expressed in all muscle fibers, whereas ACTN3 is expressed exclusively in fast glycolytic muscle fibers [14]. In humans, alpha-actinin 2 is expressed in cardiac and oxidative skeletal muscle [16].

**Figure 1:**
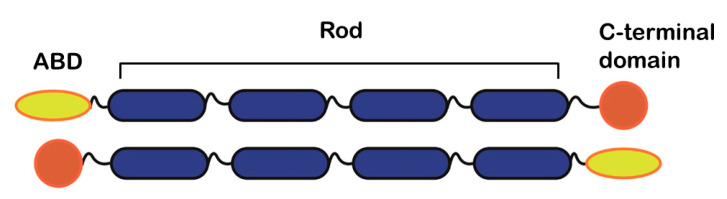
Schematic diagram illustrating the molecular organization of alpha-actinin. Relevant regions/domains (actin-binding (ABD), central rod and C-terminal domains) are highlighted.

Alpha-actinins are antiparallel dimers with a molecular mass ranging from 93 to 103 kDa [17]. The molecule is rod-shaped and has functional domains at each terminus. Specifically, the monomer consists of an actin-binding domain (ABD) at the N-terminus, followed by four spectrin repeats (the central rod) and a C-terminal calmodulin (CaM)-like domain [13] (Figure 1). 

Spectrin reiterations in the central rod are triple-helical repeats containing approximately 106 amino acid residues [17]. The rod domain acts as the rung of a ladder, establishing the distance between cross-linked actin filaments [17]. The presence of this spacer domain results in the formation of actin bundles that are loosely spaced, compared to those formed by fimbrin (which has two adjacent actin-binding domains). The link between actin and alpha-actinin was shown to be tight and strong. Indeed, the rupture forces for the actin/alpha-actinin bond were assessed in the range of 40 to 80 pN, for loading rates between 4 and 50 pN/s [18]. These data demonstrated that alpha-actinins can bear large loads while the actin cytoskeleton is deformed [19]. It is worth highlighting that alpha-actinins are not “static” elements but provide the cytoskeleton with a certain level of elasticity, for example, by exploiting unfolding and refolding events [19]. Last but not least, one isoform (ACTN1) has been reported to act as molecular shock absorber for the cytoskeleton [19], similar to lamins, which are the shock absorbers protecting the cell nucleus. This finding might suggest an analogous role (i.e., shock absorber) for the other isoforms.

As alpha-actinin 2 is the only sarcomeric isoform expressed in the heart, it has become the prime suspect in the alpha-actinins family when investigating CVDs from a mechanical perspective. Over the years, few authors made efforts to elucidate the mechanical role of alpha-actinin 2 in cardiovascular diseases. In 2012, Yang and Xu exploited transparent zebrafish embryos and performed *actn2* loss-of-function studies by antisense morpholino technology in order to assess the function of actn2 in Z-disc assembly and cardiomyopathy [16]. Depletion of actn2 was found to be crucial in sarcomere assembly only at later steps, leading to lateral alignment defects in the Z discs [16]. This primary defect resulted in the onset of cardiac-specific phenotypes in actn2 morphant embryos [16]. In particular, a reduced ventricular chamber size was detected, along with a significantly decreased cardiomyocyte size and number [16]. Further investigations suggested that smaller cardiomyocytes size might be linked to mechanical force-dependent regulation [16]. 

More recently, Lornage and colleagues presented the case of two de novo *ACTN2* mutations in two unrelated patients with progressive early-onset muscle weakness and respiratory involvement [15]. Authors studied the identified missense mutation in zebrafish and murine models, finding out that the exogenous expression of mutant ACTN2 recapitulated the abnormal muscle structure and function observed in humans [15]. In particular, sarcomeric disorganization and Z-line jagging were evident in both models [15].

## 3. Filamin C

Filamin C is a large ABP and actin crosslinking protein belonging to the wider family of filamins. In humans, it is encoded by *FLNC* gene and, in adults, is mostly expressed in cardiac and skeletal muscles [20]. In heart and muscles, FLNC localizes to Z-discs, myotendinous junctions, sarcolemma and intercalated discs [21]. Since FLNC is highly expressed in Z-discs and is structurally similar to filamin A—which has been shown to be a mechano-sensor and -transducer—it is natural to assume that filamin C is also implicated in mechanical stability, force transmission and mechanotransduction [22]. In general, filamins are key elements that crosslink actin filaments into orthogonal networks [23]. Filamin contributes to the mechanical stability of cells by linking the internal actin network with membrane receptors and mechanosensitive components. To the best of our knowledge, more than 90 different proteins—e.g., cell adhesion proteins and dystrophin-glycoprotein complex (DGC)- have been proven to interact with FLNC, but the list is being constantly broadened [21,22]. 

**Figure 2:**
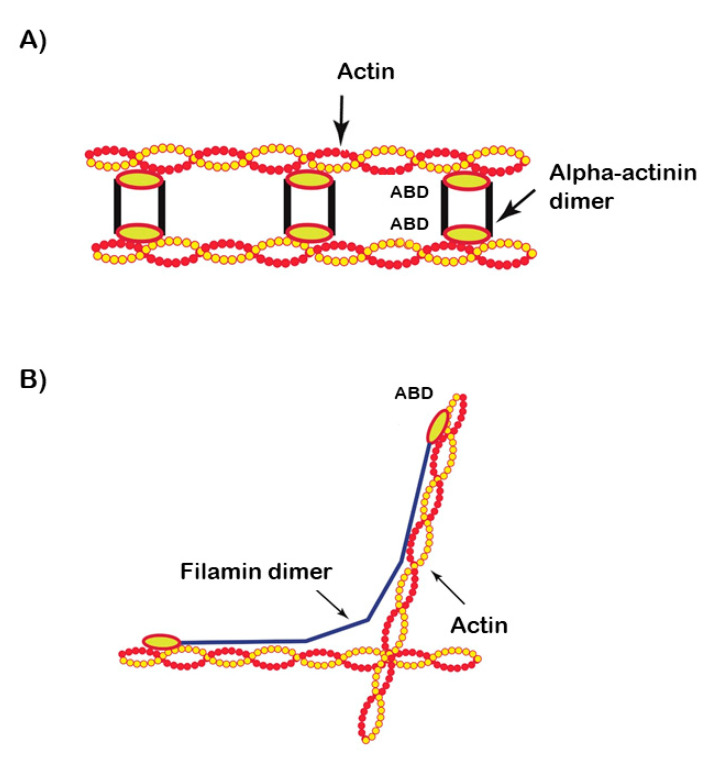
Schematic representation comparing the different microstructure and crosslinking properties of alpha-actinin (**A**) and filamin (**B**). Actin-binding domains (ABD) of both proteins are highlighted.

Filamin C has a very different microstructure compared to alpha-actinin 2 and therefore its crosslinking properties are quite distinct (Figure 2A,B).

**Figure 3:**
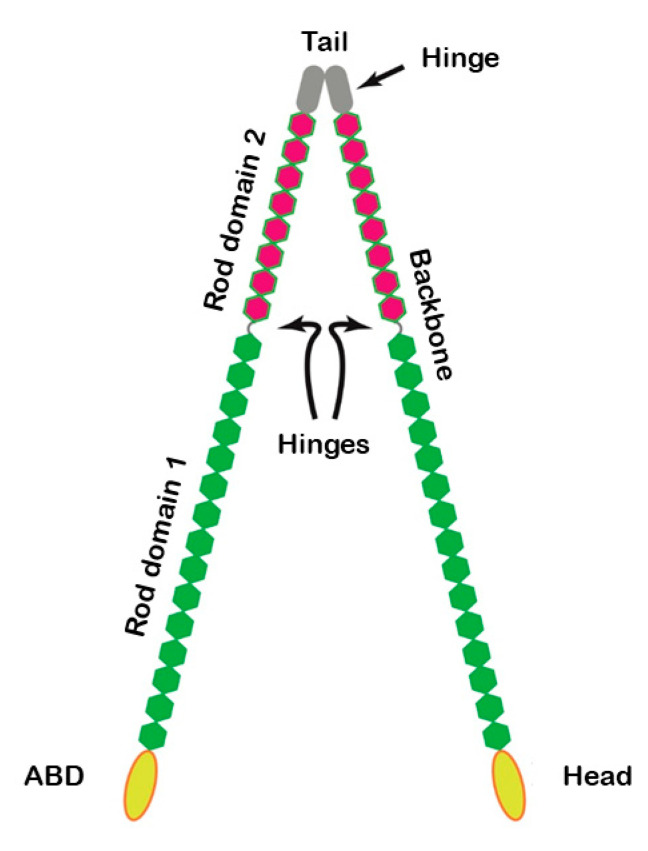
Schematic diagram illustrating the molecular organization of filamin. Relevant regions/domains, included the actin-binding domains (ABD) are highlighted.

Filamins are naturally found in cells as elongated, V-shaped dimers that dimerize at their C-termini [23]. Filamins are formed by an N-terminal spectrin-related ABD—called the “head”—followed by 24 immunoglobulin-like repeats—called the “backbone”—and a C-terminus located at the 24th repeat [22,23] (Figure 3). 

The “head” is similar to that of alpha-actinin and dystrophin and its role is the same [23]. In the “backbone” there are two hinges, that separate R15 from R16 (hinge-1) and R23 from R24 (hinge-2) and confer more flexibility to the structure [22]. Interestingly, hinge-1 has been shown to be crucial for the mechanics of actin networks. As a matter of fact, in vitro networks composed by F-actin and filamin isoforms lacking hinge-1 did not exhibit nonlinear stiffening at large stresses and broke at relatively low stress, thus never attaining the observed in vivo mechanical properties [24].

Exact structure, mechanical role and function of FLNC are yet to be fully understood but certain progresses have already been made. By way of example, FLNC has been suggested to provide mechanical support in damaged areas due to its fast recruitment to sites of injury [21]. Leber et al. exploited fluorescence recovery after photobleaching (FRAP) technique and proved that FLNC is the most dynamic sarcomeric protein since its recovery rate was very high, when compared to alpha-actinin [21]. This led to the classification of proteins of the myofibrillar Z-discs in two groups: “static” proteins, like alpha-actinin, that play a structural role and compose one group; and less integrated and “dynamic” proteins, like FLNC, that are more involved in signal transduction, maintenance and repair of the myofibril and therefore compose a different group [21]. It is also worth mentioning the recent study of Zhou and colleagues, who generated *Flnc* global knockout and cardiac-specific knockout mice to explore the specific role of FLNC in cardiomyocytes (CMs) [25]. Loss of FLNC resulted in fetal death, therefore inducible cardiomyocyte-specific knockout (icKO) mice were generated to investigate *Flnc* in adults [25]. By administering tamoxifen treatment at 2 months of age, authors ablated FLNC in icKO hearts, thus leading to the development of dilated cardiomyopathy within 2 weeks and the death of 73% of *Flnc*-icKO mice by 25 weeks [25]. Altogether, these findings demonstrated that loss of filamin C has dramatic effects on cardiomyocyte cytoskeleton [25].

## 4. Dystrophin

*DMD*, the largest known human gene, provides instructions for making a 427 kDa protein called dystrophin [26]. The *DMD* gene is located on the human chromosome Xp21 with 79 exons spanning more than 2500 kb [26]. Dystrophin is virtually present in all tissues but predominantly in skeletal muscle cells and the heart. Through the DGC, it establishes a connection between the extracellular matrix (ECM) and the actin cytoskeleton [27]. Given that, dystrophin plays an important role in maintaining cell membrane integrity and its absence can lead to membrane fragility, increase in the susceptibility to mechanical stress and abnormal permeability [26,28]. Dystrophin may also connect the Z-disc to the plasma membrane via desmin, hence contributing to lateral force transmission [27]. 

**Figure 4:**
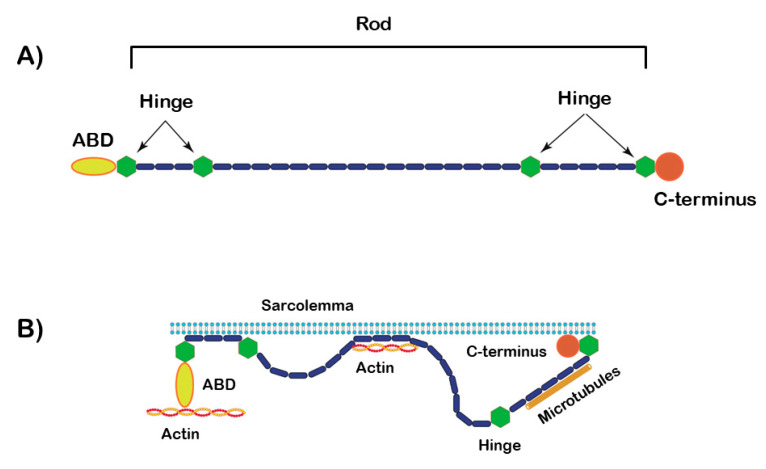
(**A**) Schematic diagram illustrating the molecular organization of dystrophin. Relevant regions/domains, including actin-binding domains (ABD), are highlighted. (**B**) shows dystrophin bonded to actin.

Since dystrophin belongs to the spectrin superfamily, it shares common features with alpha-actinins. Indeed, dystrophin consists of a N-terminal ABD, followed by a central rod domain of 24 spectrin-like repeats and a C-terminus that interacts with syntrophin and dystrobrevin [26]. The N-terminus contains a calponin-like ABD, similar to alpha-actinin [29]. As well as for alpha-actinin, the spectrin-like repeats are formed by three aligned helix bundles, which provide dystrophin with structural stiffness [29]. Despite this stiffness, the rod region shows a certain level of flexibility, resulting from breaks in the spectrin repeat pattern at four hinge regions [29]. Notably, dystrophin has a second actin-binding site located within the central rod domain (i.e., spectrin repeats 11 through 17), which cooperates with the first ABD to form a lateral association with actin network [29]. Besides the N-terminal, C-terminal and central domains, dystrophin has a fourth domain, located before the C-terminus, which is cysteine-rich and contains a WW motif, 2 EF hand motifs and ZZ motif (Figure 4A,B).

Several studies have already tried to elucidate the mechanical role of dystrophin in the cell, especially in muscle and cardiac cells. One of the oldest dates back to 1995, when Pasternak, Wong and Elson measured the contribution of dystrophin to the cortical stiffness of living muscle cells [30]. Authors used a glass stylus to indent muscle myotubes from normal and dystrophin-deficient mice, in order to assess their deformability [30]. Experiments showed that normal myotubes are stiffer than myotubes lacking dystrophin, pointing out a major role of dystrophin in the mechanical properties of muscle cell membrane skeleton [30]. The importance of the mechanical function of dystrophin has been further reported. At the cellular level, isolated, intact, murine, dystrophin-deficient cardiomyocytes were less compliant and had a greater susceptibility to stretch-mediated calcium overload respect to their normal counterparts [31]. The increased susceptibility was also found when studying the hearts of dystrophin-deficient *mdx* and control mice: abnormal hearts were less able to tolerate mechanical stress compared to normal hearts [32]. Manifestations of this susceptibility were sarcolemmal injuries and impaired contractile function [32]. These findings strengthened the mechanical role of dystrophin in the heart, by proving that cardiac dystrophin is crucial in protecting the myocardium from mechanical stress and workload-induced damage [32]. Nevertheless, additional experiments at the whole-organ level did not support the compliance data previously reported for isolated cardiomyocytes. Indeed, hearts from *mdx* mice were more compliant than controls, demonstrating an independence among whole-organ compliance, single cell compliance and sarcolemma instability [33]. Later on, transgenic mice that express Dp116 in skeletal muscles of *mdx^4cv^* mice were generated, showing that the dystrophic phenotype was exacerbated with respect to *mdx^4cv^* mice [34]. This was explained by the fact that Dp116 could reconstitute the signaling functions of dystrophin and DGC but not restore the mechanical reinforcement of the sarcolemma [34]. Authors speculated a competition mechanism between the overexpression of Dp116 and utrophin, leading to the displacement of the latter from the sarcolemma and to the further destabilization of the mechanical connection between DGC and cytoskeleton [34].

## 5. Alpha-Actinin 2 and Cardiomyopathies

Alpha-actinin 2 has been linked to cardiomyopathies more than once. *ACTN2* mutations are associated with a range of cardiac phenotypes, including but not limited to dilated cardiomyopathy (DCM) and hypertrophic cardiomyopathy (HCM) [35].

### 5.1. Hypertrophic Cardiomyopathy

Mutations in *ACTN2* have been extensively associated with hypertrophic cardiomyopathy. The latest study about this topic is the one written by Prondzynski et al., who found an *ACTN2* mutation (c.740C>T, p.T247M) in a patient with left ventricular hypertrophy, outflow tract obstruction and atrial fibrillation [36]. Authors derived human induced pluripotent stem cell-derived cardiomyocytes (hiPSC-CMs) from the patient and modified them with CRISPR to study the pathogenicity of the mutation [36]. As expected, patient derived hiPSC-CMs exhibited several hallmarks of HCM [36].

Besides this recent work, previous studies reported HCM-causing *ACTN2* mutations. The first goes back to 2006, when Theis and colleagues studied a cohort of 239 unrelated HCM patients, showing that 13 patients had one of 13 distinct HCM-associated Z-disc mutation [37]. Among them was also *ACTN2* mutation, accounting for 3 patients [37]. In 2010, Chiu and co-authors observed an Australian family exhibiting clinically heterogeneous HCM, finding a missense mutation (A119T) in *ACTN2* gene that was segregating with the disease [38]. Authors described 3 other causative *ACTN2* mutations (T495M, E583A and E628G) in 4 families with HCM, discovered among 297 HCM probands not related to the first family reported [38]. Furthermore, a large 4-generation Italian family with atypical HCM was shown to bear a disease-causing mutation in *ACTN2* [39]. Interestingly, the *ACTN2* mutation was selected among 4 likely-pathogenic variants because was the only mutation fully co-segregating with the cardiomyopathic trait [39].

In 2016, Haywood et al. investigated the mechanisms by which *ACTN2* mutations create the conditions for HCM onset. Two mutations – previously reported in association with HCM – were studied: G111V and A119T [40]. The ABD from human ACTN2, along with the mutant variants, was expressed and purified to determine structure and actin binding affinity [40]. The effects of *ACTN2* mutations were then studied in adult rat cardiomyocytes in terms of localization and dynamic behavior of full length ACTN2 [40]. Authors found out that mutations have a mild effect on ACTN2 secondary structure but decrease its stability [40]. Larger effects were observed in terms of tertiary structure and actin binding for G111V and A119T, respectively [40]. Wild type and mutant isoforms were localized to Z-discs in adult CMs but the integration of mutant ACTN2 appeared weaker and the formation of aggregates outside the Z-discs was detected in mutant samples [40]. The dynamic behavior of ACTN2 was also proved to be affected by both mutations [40]. Data collected by Haywood and colleagues collectively showed that G111V and A119T mutations affect the ability of ACTN2 to function normally, albeit leading to the onset of relatively mild clinical phenotype [40].

### 5.2. Dilated Cardiomyopathy

In the proband of one DCM family, Mohapatra et al. identified a Q9R mutation in *ACTN2* [41]. This mutation was found to be absent in controls and conserved across species [41]. Computer modeling of the mutation predicted significant changes in secondary structure and co-immunoprecipitation experiments showed a disrupted interaction between mutant ACTN2 and muscle LIM protein [41].

More recently, a novel likely-pathogenic *ACTN2* mutation (p.L320R) has been discovered in the affected members of a Chinese family with typical DCM, ventricular tachycardia, syncope and sudden cardiac death (SCD) [42].

Last, it is worth mentioning a point mutation in alpha-actinin that has been found in 2 DCM-affected Doberman Pinschers [43]. The same mutation was not detected in the other dogs of the study, therefore alpha-actinin mutation was excluded from being the exclusive causative change for DCM in Doberman Pinschers [43].

### 5.3. Arrhythmogenic Cardiomyopathy

Early in 2020, Good et al. reported the case of a Swiss family in which 4 members manifested a cardiac phenotype compatible with a left-dominant ACM [35]. By screening 10 genes involved in ACM and related cardiomyopathies (*ACTN2*, *DSG2*, *DSC2*, *PKP2*, *DSP*, *LDB3*, *JUP*, *RYR2*, *TGFβ3*, *TMEM43*), authors identified a likely-pathogenic *ACTN2* variant (p.Y473C) [35]. No other variants were observed in the panel of screened genes [35]. Although further evidence is needed to consider the mutation as disease-causing, these data shall be taken into account in the study of ACM, especially when left-dominant phenotype is manifested.

### 5.4. Others

In 2014, the group who identified the *ACTN2* mutation in the Australian family with clinically heterogeneous HCM reported the case of another Australian family with marked clinical heterogeneity, ranging from idiopathic ventricular fibrillation to left ventricular non-compaction and sudden death (SD) [44]. Authors observed the same *ACTN2* variant (A119T) they had previously identified in the other family and speculated the inheritance of the mutation from a common ancestor of both families [44]. Interestingly, the wide set of mechanistic roles of ACTN2 in the cardiac Z-disc was suggested to be accountable for the heterogeneous clinical presentations of both families [44].

## 6. Filamin C and Cardiomyopathies

A first glimpse on the impact of *FLNC* mutations in the heart is given by the study of Ortiz-Genga et al. [45]. Authors performed next-generation sequencing analyses in 2,877 patients with inherited cardiovascular diseases, finding 23 *FLNC* truncating mutations in 28 unrelated probands [45]. Among these patients, 20 were diagnosed with DCM, 7 with left-dominant ACM and 1 with RCM [45]. For *FLNC* mutations carriers, a common phenotype was identified, characterized by the overlap of DCM and ACM, plus LV systolic dysfunction and/or dilation, fibrosis, ventricular arrhythmias and a family history of SD [45]. Despite certain limitations (genetic screening limited to the genes previous associated with inherited cardiomyopathies, limited co-segregation studies because of lack of available relatives, incomplete clinical assessment in some cases, etc.), the work is certainly a milestone when studying FLNC-related cardiomyopathies [45].

### 6.1. Hypertrophic Cardiomyopathy

*FLNC* was first proposed as a potential gene implicated in HCM when whole-exome sequencing revealed a missense mutation (p.A1539T) in *FLNC* that was segregating with the disease in a HCM family [46]. This finding was confirmed by subsequent target resequencing of the candidate gene in an additional set of 92 HCM patients, which resulted in the discovery of 7 additional variants segregating with the disease in 8 families of Spanish origin [46]. Besides observing marked sarcomeric abnormalities in patients’ myocardium, filamin C aggregates were detected when expressing certain variants in H9C2 rat cardiac myoblasts, neonatal rat cardiomyocytes and C2C12 myoblasts [46]. It is worth highlighting that p.A1539T variants did not lead to the formation of FLNC aggregates in H9C2 myoblasts but caused the formation of actin aggregates, which altered the cytoskeletal structure and therefore were suspected to contribute to HCM in some families [46]. This evidence certainly strengthens the role played by filamin C in regulating cytoskeletal structure and function.

A subsequent, large-scale screening of *FLNC* in a HCM cohort confirmed that *FLNC* variants are likely associated with HCM development [47]. 448 HCM patients and 450 healthy controls were screened; by taking into account population frequencies, bioinformatics prediction, functional studies and familial segregation, 6 candidate variants were identified as likely-pathogenic, 10 variants as of uncertain significance, 4 variants as likely-benign [47]. Nonetheless, when considering the American College of Medical Genetics and Genomics criteria, only 2 variants (namely p.V123A and p.A1539T) remained likely-pathogenic [47].

Further *FLNC* variants related to HCM are available for reference in the papers of Jafaar et al. and Verdonschot et al. [48,49].

### 6.2. Dilated Cardiomyopathy

Up to now, *FLNC* mutations are recognized as one of the most common cause of genetic DCM [49]. In 2016, Begay et al. reported *FLNC* splicing variants by studying 3 families (two from Italy, one from United States (US)), in which affected members were diagnosed with DCM plus a strong arrhythmogenic trait [50]. In the Italian families, *FLNC* c.7251+1G>A variant was identified, whereas in the US family the variant discovered was *FLNC* c.5669-1delG [50]. Both variants caused a reduction of cardiac FLNC protein levels, suggesting a haploinsufficiency model [50]. Haploinsufficiency was further supported by the data collected with morpholino knockdown of *flncb* in zebrafish embryos [50]. Mutant embryos generally exhibited cardiac phenotype, characterized by structural abnormalities in Z-discs and lower survival at 7 days post fertilization respect to control embryos [50].

Arrhythmogenic DCM and *FLNC* mutations had been associated again in the study that identified 6 *FLNC* truncating mutations in 7 out of 319 DCM families [51]. The observed cellular phenotype partially overlapped with ACM, and interstitial fibrosis was detected in the RV, alongside epicardial fibrofatty infiltration in the LV, occasional disarray of Z-discs in the sarcomere and decreased signals for desmoplakin and synapse-associated protein 97 in the myocardium and buccal mucosa [51]. The phenotypes overlap was ascribed to the suggested haploinsufficiency model, which induced a range of events (disrupted crosslinking function, abnormal connection of actin to cell-cell adhesion, etc.), eventually resulting in the interference with the desmosomal/cell–cell junction pathway and the onset of ACM phenotype [51].

Finally, in an Iranian family with a history of DCM spanning over 4 generations, a novel splicing *FLNC* variant (c.2389+1 G>A) had been identified [52]. Among carriers, DCM severity and age of disease onset were variable and most frequent symptoms were exertional dyspnea and palpitations [52]. The study reported 11 affected family members but 6 more individuals could have been carriers, since they died of sudden cardiac death before 65 years old [52].

### 6.3. Arrhythmogenic Cardiomyopathy

The first characterization of *FLNC* variants in ACM has been recently proposed by Hall and colleagues, who identified 4 null *FLNC* variants and 5 *FLNC* variants of unknown significance [53]. Because of limited information on pedigrees and ambiguous pathogenicity, authors focused their study on the families carrying null variants, excluding variants of unknown significance [53]. Significant phenotypic differences were observed between classic desmosomal ARVC and ACM associated with null *FLNC* variants [53]. Among them is the absence of the mislocalization of plakoglobin or GSKβ3 [53]. Conversely, immunohistochemistry revealed that FLNC signal was reduced in the left ventricle and magnetic resonance imaging (MRI) showed late gadolinium enhancement (LGE) with preserved ventricular function [53]. LGE was detected also in the majority of gene positive family members and this evidence contributed to define these family members as at-risk individuals [53]. This is a very interesting result, since LGE analysis is not currently included in the criteria for ACM diagnosis, therefore family members could have been incorrectly classified [53].

Earlier in 2020, more *FLNC* variants have been identified in two unrelated families as a novel cause of an ACM phenotype characterized by predominant RV involvement and life-threatening ventricular arrhythmias [54]. Among 156 ARVC patients, Brun et al. found two *FLNC* variants (c.6565 G>T and c.8107delG) that were classified as pathogenic according to the American College of Medical Genetics criteria [54].

### 6.4. Restrictive Cardiomyopathy

A Canadian study was the first to show the link between restrictive cardiomyopathy and *FLNC* mutations [55]. In two Caucasian families with autosomal-dominant RCM, two novel missense *FLNC* variants were identified (c.4871C>T (p.S1624L) and c.6478A>T (p.I2160F)) [55]. Immunohistochemistry on cardiac tissues from both families showed contrasting data: in the family bearing p.S1624L mutation, filamin C aggregates and disturbed Z-disc staining were clearly detected, whereas in the other family (p.I2160F), filamin C aggregates were not found [55]. Conversely, in cardiac tissues of both families the intercalated disc localization of desmin was absent [55]. Observations in patients were supported by transfecting rat and mouse myoblasts (namely H9C2 and C2C12 cell lines) with mutant FLNC [55]. p.S1624L variant led to the formation of protein aggregates, whilst p.I2160F variant was not abnormally localized respect to the wild type counterpart [55]. Overall, this study demonstrated a likely-pathogenicity of p.S1624L mutation and a likely involvement of p.I2160F variant in RCM onset [55].

A single novel missense variant (c.6889 G>A (p.V2297M)) was also identified as a potential cause of RCM in a 4-generation family, being the only variant shared by all affected members and absent from control datasets [56]. In the explanted heart of one family member, filamin C was localized to the intercalated disc but the association of FLNC with the sarcomeric architecture was remarkably reduced respect to the controls [56]. In C2C12 myoblasts transfected with either wild-type or mutant FLNC, filamin C localized to the stress fiber architecture, thus demonstrating that the actin-binding ability was not affected by the mutation [56]. The measurement of contractile activity of wild-type and mutant stem cell-derived cardiomyocytes showed an impairment of the contractile apparatus caused by the mutant protein [56]. Altogether, these results encouraged to propose p.V2297M as a pathogenic variant according the guidelines of the American College of Medical Genetics and Genomics [56].

Two more *FLNC* mutations have been reported in RCM families with childhood onset—c.6893C>T (p.P2298L) and c.7688A>G (p.Y2563C) [57]. The study of explanted cardiac tissue in the family with p.P2298L variant showed no inclusions or depositions and the sarcomere structure presented regular and without disarrays [57]. Same conclusions were drawn from the explanted hearts in the family with p.Y2563C mutation [57]. Conversely, the expression of filamin C variants in C2C12 cells led to aggregate formation [57]. Specifically, in cells expressing p.P2298L construct, aggregates were often observed in the proximity of nuclei [57]. On the other hand, C2C12 cells with p.Y2563C variant exhibited more punctate and varied in size—from (from small to intermediate—FLNC) FLNC aggregates [57]. Interestingly, both variants resulted in the formation of aggregates of both mutant FLNC and F-actin [57].

Finally, filamin C mutations have been reported also in association with the early onset of severe RCM in combination with congenital myopathy [58]. p.A1183L and p.A1186V variants were indeed identified in four patients [58]. While absence of protein aggregates was shown in C2C12 cells transfected with both variants, in vivo studies of zebrafish model revealed the presence of filamin C aggregates due to overexpression of mutant FLNCs [58]. Despite sharing the same genetic background, FLNC aggregates were detected solely in the oldest patient examined but not in the youngest, therefore authors suggested that aggregates formation could be ascribed to the intracellular protein degradation system [58].

## 7. Dystrophin and Cardiomyopathies

Before discussing the effects of dystrophin mutations in the heart, a brief introduction for dystrophinopathies shall be provided. Dystrophinopaties are muscular disorders characterized by X-linked inheritance pattern and include Duchenne muscular dystrophy (DMD), Becker muscular dystrophy (BMD) and X-linked dilated cardiomyopathy (XLDCM). In DMD and BMD, skeletal muscles are primarily affected, although cardiac muscles can be involved too. In fact, cardiomyopathies are becoming an increasingly recognized manifestation of DMD and BMD, mainly because they significantly contribute to their morbidity and mortality [59]. Almost all DMD patients suffer from cardiomyopathy after age 18 years and the most common cause of death for BMD individuals is heart failure from DCM [60]. Cardiomyopathies secondary to dystrophinopathies have variable phenotypes that are likely resulting from the mechanical stress on a metabolically and structurally abnormal myocardium [61].

This work is focused only on XLDCM as usually it is cardiac-specific, without overt signs of skeletal disorders.

### X-Linked Dilated Cardiomyopathy

As mentioned before, X-linked dilated cardiomyopathy (XLDCM) is a distinctive phenotype of dystrophinopathy characterized by preferential cardiac involvement without evident skeletal manifestations. In 1987, Berko and Swift reported the case of a large kindred in which they had recognized an early expression and rapid progression of cardiomyopathy in males, later onset with slower progression in females and no male-to-male transmission of a cardiac disorder [62]. Their observations resulted in the final description of an X-linked form of DCM [62].

To the best of our knowledge, the first connection between *DMD* and XLDCM dates back to 1993, when Towbin et al. published a groundbreaking study showing that two families with X-linked DCM carried a genetic defect in the dystrophin locus within chromosome region Xp21 [63]. One year later (1994), the case of a 14 year-old boy with mild muscular weakness and severe signs of cardiac dysfunction was reported [64]. The patient died of DCM shortly after his referral and before getting cardiac transplantation [64]. Later examinations showed an unusual deletion of the dystrophin gene, causing a shortening of the encoded protein near the N-terminus [64]. The case was classified as “sporadic case of DCM” because authors were not able to unequivocally identify the mother as the carrier mutation [64]. Conversely to the aforementioned patient, the 16-years old patient reported by Lester and coworkers, who had no cardiac history but presented with left-sided weakness and facial droop, successfully underwent cardiac transplantation after being diagnosed with XLDCM [61]. In this case, the X-linked inheritance pattern was established by finding out that an estranged maternal uncle was diagnosed with DCM and had an X-linked dystrophin gene mutation [61]. Besides the latter work, heart transplantation success in XLDCM patients was disclosed by an Italian group too. Specifically, seven patients with age ranging from 16 to 31 years and diagnosed with end-stage XLDCM underwent heart transplantation between August 1989 and January 2000 [65]. Since only one patient among them suddenly died at 66 months follow-up, heart transplantation was advised by the authors as a life-saving procedure for end-stage XLDCM patients [65]. Another positive outcome of heart transplantation in a 27-years old XLDCM patient is mentioned in Reference [66].

Up to now, we cited studies mostly about adolescents but cases of older patients have been also presented. By way of example, we report the study of Tasaki and colleagues, describing a previously healthy 36-years-old man, who was later diagnosed with XLDCM according to clinical, laboratory and molecular genetic findings [67]. X-linked inheritance pattern was confirmed as the mother was diagnosed as heterozygous carrier [67].

Lastly, it is worth mentioning the recent study of Chamberlain et al. A young boy—16-years old—suffered from palpitations progressing to heart failure, with no evident clinically skeletal muscle weakness [68]. Due to the elevated total serum creatine kinase level—typical in XLDCM—the dystrophin gene comparative genomic hybridization array was submitted, finding out a mutation in both the patient and his mother [68]. Of note, authors reported that the family had declined the testing for the patients’ brothers [68].

Other details for dystrophin-related XLDCM can be found in the extensive work of Nakamura [26].

## 8. Conclusions

Cardiovascular disorders are a major cause of death worldwide and inherited cardiomyopathies represent an important epidemiological entity. Despite the strenuous efforts made in cardiology, early diagnosis and resolutive therapies are still often lacking. In order to better understand the pathogenesis of cardiomyopathies, multiple approaches shall be pursued. Among them is the study of structural and mechanical properties of biological systems, like cells, tissues and organs, because knowledge of these characteristics has been shown to be requisite for understanding causes and consequences of heart dysfunction.

In this regard, investigating how events at very small scale like genetic mutations can impair the cytoskeletal organization and eventually cause events at larger scale like cardiomyopathies may open new routes for creating novel diagnostic tools and more specific therapies. To this aim, we have provided a collection of studies that show the connection between mutant cytoskeletal cross-linkers and inherited cardiomyopathies. In particular, we have focused on actin-binding and crosslinking proteins, that is alpha-actinin 2, filamin C and dystrophin, because actin has a major role in the cell, being the most abundant intracellular protein. We have described the structure, mechanical properties, similarities and differences of the aforementioned proteins and then reported the cardiac pathologic phenotypes found to be associated—or likely associated—with mutations in their coding genes. We have recalled that all these cross-linkers are crucial for regulating the properties of actin cytoskeleton and the cytoskeleton itself—in fact, networks composed by actin and filamin lacking hinge-1 are not mechanically comparable to in vivo structures and FLNC variants can lead to the formation of actin aggregates. The observation of these phenomena at the smallest scales might inform future endeavors in new therapeutic opportunities.

Through our work, we have showed that, despite sharing the common role of actin-binding and crosslinking proteins, *ACTN2*, *FLNC* and *DMD* mutations can cause different types of cardiomyopathies, ranging from HCM to RCM. The heterogeneity of pathologic phenotypes is not limited to the differences among these proteins but is manifested even within the same coding gene (i.e., distinct mutations in a single gene (e.g., *ACTN2*) are causative of multiple pathologies (e.g., HCM, DCM and ACM)).

The identification of mutations in patients with cardiomyopathies is pivotal to establish diagnosis and treatment and together with biomarkers of myocardial failure, such as brain natriuretic peptide (BNP) and N-terminal proBNP (NT-proBNP), can represent the starting point for implementing a *precision medicine* approach. Moreover, finding causative genetic mutations in cardiomyopathy patients is the main driving force for the genetic counseling of family members who could be individuals at risk. Hereby, we have reported what has already been discovered about ACTN2, FLNC and dystrophin in inherited cardiomyopathies and our effort might set the stage for broadening the panel of screened genes in cardiomyopathy patients by including the genes that code for other cytoskeletal cross-linkers. Among potential candidates for the extended panel, worthy of special mention are nesprins, in particular nesprin-1. Nesprins were the latest members of the spectrin-repeat super family to be identified and as well as the proteins discussed in this review, they possess a N-terminal actin-binding domain followed by a rod-like structure formed by spectrin repeats [69]. Similarities with ACTN2, FLNC and dystrophin go beyond the structure—indeed, nesprin-1 mutations had been already associated with the onset of DCM [70,71]. Although the function of nesprins as cytoskeletal cross-linkers may be questionable so far, their resemblance with ACTN2, FLNC and dystrophin and their involvement in cardiac pathology should prompt extensive research to elucidate their role in cell structure and discover other pathologic phenotypes besides DCM.

## Figures and Tables

**Table 1:** Schematic overview of the characteristics of certain cardiomyopathies hereby discussed.

Type	Brief Clinical Description
Hypertrophic cardiomyopathy (HCM)	HCM is characterized by cardiac hypertrophy in the absence of secondary causes; nondilated left ventricle (LV); normal or increased ejection fraction. Hypertrophy is typically asymmetric, mainly affecting the basal interventricular septum subjacent to the aortic valve [6]
Dilated cardiomyopathy (DCM)	Dilation and impaired contraction of left ventricle or both ventricles characterize DCM. LV systolic dysfunction is variable and often progressive [7,8]
Arrhythmogenic cardiomyopathy (ACM)	The term “ACM” encompasses a broad range of phenotypic expressions, including arrhythmogenic right/left ventricular cardiomyopathy (ARVC, ALVC), cardiac amyloidosis, sarcoidosis, Chagas disease and left ventricular noncompaction. The original disease phenotype (i.e., ARVC) is characterized by prominent ventricular arrhythmias (with predominant right ventricle involvement) and impairment of ventricular systolic function [9,10]
Restrictive cardiomyopathy (RCM)	Distinctive features of RCM are increased myocardial stiffness, impaired ventricular filling, and impaired diastolic function. Systolic function is usually preserved or near-normal, until later stages of the disease. Wall thickness is generally normal or mildly increased. Arrhythmias and conduction disturbances are often observed [11,12]

## References

[1] Girolami F., Frisso G., Benelli M., Crotti L., Iascone M., Mango R., Mazzaccara C., Pilichou K., Arbustini E., Tomberli B. (2018). Contemporary genetic testing in inherited cardiac disease. J. Cardiovasc. Med..

[2] Asatryan B., Marcus F.I. (2020). The Ever-Expanding Landscape of Cardiomyopathies. JACC Case Rep..

[3] McKenna W.J., Maron B.J., Thiene G. (2017). Classification, Epidemiology, and Global Burden of Cardiomyopathies. Circ. Res..

[4] Egan P.F., Sinko R., LeDuc P.R., Keten S. (2015). The role of mechanics in biological and bio-inspired systems. Nat. Commun..

[5] Fletcher D.A., Mullins R.D. (2010). Cell mechanics and the cytoskeleton. Nature.

[6] Marian A.J., Braunwald E. (2017). Hypertrophic Cardiomyopathy: Genetics, Pathogenesis, Clinical Manifestations, Diagnosis, and Therapy. Circ. Res..

[7] Mestroni L., Maisch B., McKenna W.J., Schwartz K., Charron P., Rocco C., Tesson F., Richter R., Wilke A., Komajda M. (1999). Guidelines for the study of familial dilated cardiomyopathies. Eur. Heart J..

[8] Oni A.A., Hosenpud J.D. (1991). Dilated cardiomyopathy. Curr. Opin. Cardiol..

[9] Towbin J.A., McKenna W.J., Abrams M.J., Ackerman M.J., Calkins H., Darrieux F.C., Daubert J.P., De Chillou C., DePasquale E.C., Desai M.Y. (2019). 2019 HRS expert consensus statement on evaluation, risk stratification, and management of arrhythmogenic cardiomyopathy. Heart Rhythm..

[10] Corrado D., Basso C., Judge D.P. (2017). Arrhythmogenic Cardiomyopathy. Circ. Res..

[11] Muchtar E., Blauwet L.A., Gertz M.A. (2017). Restrictive Cardiomyopathy: Genetics, Pathogenesis, Clinical Manifestations, Diagnosis, and Therapy. Circ. Res..

[12] Rammos A., Meladinis V., Vovas G., Patsouras D. (2017). Restrictive Cardiomyopathies: The Importance of Noninvasive Cardiac Imaging Modalities in Diagnosis and Treatment—A Systematic Review. Radiol. Res. Pr..

[13] Sjöblom B., Salmazo A., Carugo K.D. (2008). α-Actinin structure and regulation. Cell. Mol. Life Sci..

[14] Lek M., North K.N. (2010). Are biological sensors modulated by their structural scaffolds? The role of the structural muscle proteins α-actinin-2 and α-actinin-3 as modulators of biological sensors. FEBS Lett..

[15] Lornage X., Romero N.B., Grosgogeat C.A., Malfatti E., Donkervoort S., Marchetti M.M., Neuhaus S.B., Foley A.R., Labasse C., Schneider R. (2019). ACTN2 mutations cause “Multiple structured Core Disease” (MsCD). Acta Neuropathol..

[16] Yang J., Xu X. (2012). α -Actinin2 is required for the lateral alignment of Z discs and ventricular chamber enlargement during zebrafish cardiogenesis. FASEB J..

[17] Virel A., Backman L. (2004). Molecular Evolution and Structure of α-Actinin. Mol. Biol. Evol..

[18] Ferrer J.M., Lee H., Chen J., Pelz B., Nakamura F., Kamm R.D., Lang M.J. (2008). Measuring molecular rupture forces between single actin filaments and actin-binding proteins. Proc. Natl. Acad. Sci. USA.

[19] Le S., Hu X., Yao M., Chen H., Yu M., Xu X., Nakazawa N., Margadant F., Sheetz M.P., Yan J. (2017). Mechanotransmission and Mechanosensing of Human alpha-Actinin 1. Cell Rep..

[20] Feng Y., Walsh C.A. (2004). The many faces of filamin: A versatile molecular scaffold for cell motility and signalling. Nat. Cell Biol..

[21] Leber Y., Ruparelia A.A., Kirfel G., Van Der Ven P.F., Hoffmann B., Merkel R., Bryson-Richardson R.J., Fürst D.O. (2016). Filamin C is a highly dynamic protein associated with fast repair of myofibrillar microdamage. Hum. Mol. Genet..

[22] Mao Z., Nakamura F. (2020). Structure and Function of Filamin C in the Muscle Z-Disc. Int. J. Mol. Sci..

[23] Stossel T.P., Condeelis J., Cooley L., Hartwig J.H., Noegel A., Schleicher M., Shapiro S.S. (2001). Filamins as integrators of cell mechanics and signalling. Nat. Rev. Mol. Cell Biol..

[24] Gardel M.L., Nakamura F., Hartwig J.H., Crocker J.C., Stossel T.P., Weitz D.A. (2006). Prestressed F-actin networks cross-linked by hinged filamins replicate mechanical properties of cells. Proc. Natl. Acad. Sci. USA.

[25] Zhou Y., Chen Z., Zhang L., Zhu M., Tan C., Zhou X., Evans S.M., Fang X., Feng W., Chen J. (2020). Loss of Filamin C Is Catastrophic for Heart Function. Circulation.

[26] Nakamura A. (2015). X-Linked Dilated Cardiomyopathy: A Cardiospecific Phenotype of Dystrophinopathy. Pharmaceuticals.

[27] Dobner S., Amadi O.C., Lee R.T. (2012). Cardiovascular Mechanotransduction. Muscle.

[28] Escolar D.M., O’Carroll P., Leshner R. (2011). Treatment and Management of Muscular Dystrophies. Neuromuscular Disorders: Treatment and Management.

[29] Lapidos K.A., Kakkar R., McNally E.M. (2004). The Dystrophin Glycoprotein Complex: Signaling Strength and Integrity for the Sarcolemma. Circ. Res..

[30] Pasternak C., Wong S., Elson E.L. (1995). Mechanical function of dystrophin in muscle cells. J. Cell Biol..

[31] Yasuda S., Townsend D., Michele D.E., Favre E.G., Day S.M., Metzger J.M. (2005). Dystrophic heart failure blocked by membrane sealant poloxamer. Nature.

[32] Danialou G., Comtois A.S., Dudley R., Karpati G., Vincent G., Rosiers C.D., Petrof B.J. (2001). Dystrophin-deficient cardiomyocytes are abnormally vulnerable to mechanical stress-induced contractile failure and injury. FASEB J..

[33] Barnabei M.S., Metzger J.M. (2012). Ex Vivo Stretch Reveals Altered Mechanical Properties of Isolated Dystrophin-Deficient Hearts. PLoS ONE.

[34] Judge L.M., Haraguchiln M., Chamberlain J.S. (2006). Dissecting the signaling and mechanical functions of the dystrophin-glycoprotein complex. J. Cell Sci..

[35] Good J.-M., Fellmann F., Bhuiyan Z.A., Rotman S., Pruvot E., Schläpfer J. (2020). ACTN2 variant associated with a cardiac phenotype suggestive of left-dominant arrhythmogenic cardiomyopathy. Heart Case Rep..

[36] Prondzynski M., Lemoine M.D., Zech A.T., Horváth A., Di Mauro V., Koivumäki J.T., Kresin N., Busch J., Krause T., Krämer E. (2019). Disease modeling of a mutation in α-actinin 2 guides clinical therapy in hypertrophic cardiomyopathy. EMBO Mol. Med..

[37] Theis J.L., Bos J.M., Bartleson V.B., Will M.L., Binder J., Vatta M., A Towbin J., Gersh B.J., Ommen S.R., Ackerman M.J. (2006). Echocardiographic-determined septal morphology in Z-disc hypertrophic cardiomyopathy. Biochem. Biophys. Res. Commun..

[38] Chiu C.L., Bagnall R.D., Ingles J., Yeates L., Kennerson M., Donald J., Jormakka M., Lind J.M., Semsarian C. (2010). Mutations in Alpha-Actinin-2 Cause Hypertrophic Cardiomyopathy. J. Am. Coll. Cardiol..

[39] Girolami F., Iascone M., Tomberli B., Bardi S., Benelli M., Marseglia G., Pescucci C., Pezzoli L., Sana M.E., Basso C. (2014). Novel α-Actinin 2 Variant Associated With Familial Hypertrophic Cardiomyopathy and Juvenile Atrial Arrhythmias: A Massively Parallel Sequencing Study. Circ. Cardiovasc. Genet..

[40] Haywood N.J., Wolny M., Rogers B., Trinh C.H., Shuping Y., Edwards T.A., Peckham M. (2016). Hypertrophic cardiomyopathy mutations in the calponin-homology domain of ACTN2 affect actin binding and cardiomyocyte Z-disc incorporation. Biochem. J..

[41] Mohapatra B., Jimenez S., Lin J.H., Bowles K.R., Coveler K.J., Marx J.G., Chrisco M.A., Murphy R.T., Lurie P.R., Schwartz R.J. (2003). Mutations in the muscle LIM protein and α-actinin-2 genes in dilated cardiomyopathy and endocardial fibroelastosis. Mol. Genet. Metab..

[42] Fan L.-L., Huang H., Jin J.-Y., Li J.-J., Chen Y.-Q., Xiang R. (2019). Whole-Exome Sequencing Identifies a Novel Mutation (p.L320R) of Alpha-Actinin 2 in a Chinese Family with Dilated Cardiomyopathy and Ventricular Tachycardia. Cytogenet. Genome Res..

[43] O’Sullivan M.L., O’Grady M.R., Pyle W.G., Dawson J.F. (2011). Evaluation of 10 genes encoding cardiac proteins in Doberman Pinschers with dilated cardiomyopathy. Am. J. Veter. Res..

[44] Bagnall R.D., Molloy L.K., Kalman J.M., Semsarian C. (2014). Exome sequencing identifies a mutation in the ACTN2 gene in a family with idiopathic ventricular fibrillation, left ventricular noncompaction, and sudden death. BMC Med. Genet..

[45] Ortiz-Genga M., Cuenca S., Ferro M.D., Zorio E., Salgado-Aranda R., Climent V., Padrón L., Duro-Aguado I., Jiménez-Jáimez J., Hidalgo-Olivares V.M. (2016). Truncating FLNC Mutations Are Associated With High-Risk Dilated and Arrhythmogenic Cardiomyopathies. J. Am. Coll. Cardiol..

[46] Valdés-Mas R., Gutierrez-Fernandez A., Gómez J., Coto E., Astudillo A., Puente D.A., Reguero J.R., Alvarez V., Morís C., León D. (2014). Mutations in filamin C cause a new form of familial hypertrophic cardiomyopathy. Nat. Commun..

[47] Gómez J., Lorca R., Reguero J.R., Morís C., Martín M., Tranche S., Alonso B., Iglesias S., Alvarez V., Díaz-Molina B. (2017). Screening of the Filamin C Gene in a Large Cohort of Hypertrophic Cardiomyopathy Patients. Circ. Cardiovasc. Genet..

[48] Jaafar N., Gómez J., Kammoun I., Zairi I., Ben Amara W., Kachboura S., Kraiem S., Hammami M., Iglesias S., Alonso B. (2016). Spectrum of Mutations in Hypertrophic Cardiomyopathy Genes Among Tunisian Patients. Genet. Test. Mol. Biomark..

[49] Verdonschot J., Vanhoutte E.K., Claes G.R.F., Enden A.T.J.M.H.D., Hoeijmakers J.G.J., Hellebrekers D.M.E.I., De Haan A., Christiaans I., Deprez R.H.L., Boen H.M. (2020). A mutation update for the FLNC gene in myopathies and cardiomyopathies. Hum. Mutat..

[50] Begay R.L., Tharp C.A., Martin A., Graw S.L., Sinagra G., Miani D., Sweet M.E., Slavov D.B., Stafford N., Zeller M.J. (2016). FLNC Gene Splice Mutations Cause Dilated Cardiomyopathy. JACC Basic Transl. Sci..

[51] Begay R.L., Graw S.L., Sinagra G., Asimaki A., Rowland T.J., Slavov D.B., Gowan K., Jones K.L., Brun F., Merlo M. (2018). Filamin C Truncation Mutations Are Associated With Arrhythmogenic Dilated Cardiomyopathy and Changes in the Cell-Cell Adhesion Structures. JACC Clin. Electrophysiol..

[52] Nozari A., Aghaei-Moghadam E., Zeinaloo A., Mollazadeh R., Majnoon M.-T., Alavi A., Firouzabadi S.G., Mohammadzadeh A., Banihashemi S., Nikzaban M. (2018). A novel splicing variant in FLNC gene responsible for a highly penetrant familial dilated cardiomyopathy in an extended Iranian family. Gene.

[53] Hall C.L., Akhtar M.M., Sabater-Molina M., Futema M., Asimaki A., Protonotarios A., Dalageorgou C., Pittman A.M., Suarez M.P., Aguilera B. (2020). Filamin C variants are associated with a distinctive clinical and immunohistochemical arrhythmogenic cardiomyopathy phenotype. Int. J. Cardiol..

[54] Brun F., Gigli M., Graw S.L., Judge D.P., Merlo M., Murray B., Calkins H., Sinagra G., Taylor M.R., Mestroni L. (2020). FLNC truncations cause arrhythmogenic right ventricular cardiomyopathy. J. Med Genet..

[55] Brodehl A., Ferrier R.A., Hamilton S.J., Greenway S.C., Brundler M.-A., Yu W., Gibson W.T., McKinnon M.M., McGillivray B., Alvarez N. (2016). Mutations inFLNCare Associated with Familial Restrictive Cardiomyopathy. Hum. Mutat..

[56] Tucker N.R., McLellan M.A., Hu D., Ye J., Parsons V.A., Mills R.W., Clauss S., Dolmatova E., Shea M.A., Milan D.J. (2017). Novel Mutation in FLNC (Filamin C) Causes Familial Restrictive Cardiomyopathy. Circ. Cardiovasc. Genet..

[57] Schubert J., Tariq M., Geddes G., Kindel S., Miller E.M., Ware S.M. (2018). Novel pathogenic variants in filamin C identified in pediatric restrictive cardiomyopathy. Hum. Mutat..

[58] Kiselev A., Vaz R., Knyazeva A., Khudiakov A., Tarnovskaya S., Liu J., Sergushichev A., Kazakov S., Frishman D., Smolina N.A. (2018). De novo mutations in FLNC leading to early-onset restrictive cardiomyopathy and congenital myopathy. Hum. Mutat..

[59] Kamdar F., Garry D.J. (2016). Dystrophin-Deficient Cardiomyopathy. J. Am. Coll. Cardiol..

[60] Brandsema J.F., Darras B.T. (2015). Dystrophinopathies. Semin. Neurol..

[61] Lester G., Femia G., Ayer J., Puranik R. (2019). A case report: X-linked dystrophin gene mutation causing severe isolated dilated cardiomyopathy. Eur. Heart J. Case Rep..

[62] Berko B.A., Swift M. (1987). X-Linked Dilated Cardiomyopathy. N. Engl. J. Med..

[63] Towbin J.A., Hejtmancik J.F., Brink P., Gelb B., Zhu X.M., Chamberlain J.S., McCabe E.R., Swift M. (1993). X-linked dilated cardiomyopathy. Molecular genetic evidence of linkage to the Duchenne muscular dystrophy (dystrophin) gene at the Xp21 locus. Circulation.

[64] Oldfors A., Eriksson B.O., Kyllerman M., Martinsson T., Wahlstrom J. (1994). Dilated cardiomyopathy and the dystrophin gene: An illustrated review. Heart.

[65] Grande A.M., Rinaldi M., Pasquino S., D’Armini A.M., Viganò M. (2002). Heart transplantation in X-linked dilated cardiomyopathy. Ital. Heart J. Off. J. Ital. Fed. Cardiol..

[66] Papa A.A., D’Ambrosio P., Petillo R., Palladino A., Politano L. (2017). Heart transplantation in patients with dystrophinopathic cardiomyopathy: Review of the literature and personal series. Intractable Rare Dis. Res..

[67] Tasaki N., Yoshida K., Haruta S.-I., Kouno H., Ichinose H., Fujimoto Y., Urasawa N., Kawakami T., Taniguchi M., Kurushima S.-J. (2001). X-linked Dilated Cardiomyopathy with a Large Hot-spot Deletion in the Dystrophin Gene. Intern. Med..

[68] Chamberlain R.C., Smith E.C., Campbell M.J. (2015). Novel Rod Domain Duplication in Dystrophin Resulting in X-Linked Dilated Cardiomyopathy. Pediatr. Neurol..

[69] Rajgor D., Shanahan C.M. (2013). Nesprins: From the nuclear envelope and beyond. Expert Rev. Mol. Med..

[70] Puckelwartz M.J., Kessler E.J., Kim G., DeWitt M.M., Zhang Y., Earley J.U., Depreux F.F., Holaska J.M., Mewborn S.K., Pytel P. (2010). Nesprin-1 mutations in human and murine cardiomyopathy. J. Mol. Cell. Cardiol..

[71] Zhou C., Li C., Zhou B., Sun H., Koullourou V., Holt I., Puckelwartz M.J., Warren D.T., Hayward R., Lin Z. (2017). Novel nesprin-1 mutations associated with dilated cardiomyopathy cause nuclear envelope disruption and defects in myogenesis. Hum. Mol. Genet..

